# Development of Robust Freeze-Drying Process for Long-Term Stability of rVSV-SARS-CoV-2 Vaccine

**DOI:** 10.3390/v16060942

**Published:** 2024-06-11

**Authors:** MD Faizul Hussain Khan, Maryam Youssef, Sean Nesdoly, Amine A. Kamen

**Affiliations:** Viral Vectors and Vaccines Bioprocessing Group, Department of Bioengineering, McGill University, Montreal, QC H2X 1Y4, Canada; md.f.khan@mail.mcgill.ca (M.F.H.K.);

**Keywords:** vesicular stomatitis virus, enveloped viral vector vaccine, freeze-drying, solid formulation, stability, viral vaccine bioprocess, VSV, CPPs, CQAs, rVSV-SARS-CoV-2, COVID-19

## Abstract

The thermostability of vaccines, particularly enveloped viral vectored vaccines, remains a challenge to their delivery wherever needed. The freeze-drying of viral vectored vaccines is a promising approach but remains challenging due to the water removal process from the outer and inner parts of the virus. In the case of enveloped viruses, freeze-drying induces increased stress on the envelope, which often leads to the inactivation of the virus. In this study, we designed a method to freeze-dry a recombinant vesicular stomatitis virus (VSV) expressing the SARS-CoV-2 spike glycoprotein. Since the envelope of VSV is composed of 50% lipids and 50% protein, the formulation study focused on both the protein and lipid portions of the vector. Formulations were prepared primarily using sucrose, trehalose, and sorbitol as cryoprotectants; mannitol as a lyoprotectant; and histidine as a buffer. Initially, the infectivity of rVSV-SARS-CoV-2 and the cake stability were investigated at different final moisture content levels. High recovery of the infectious viral titer (~0.5 to 1 log loss) was found at 3–6% moisture content, with no deterioration in the freeze-dried cakes. To further minimize infectious viral titer loss, the composition and concentration of the excipients were studied. An increase from 5 to 10% in both the cryoprotectants and lyoprotectant, together with the addition of 0.5% gelatin, resulted in the improved recovery of the infectious virus titer and stable cake formation. Moreover, the secondary drying temperature of the freeze-drying process showed a significant impact on the infectivity of rVSV-SARS-CoV-2. The infectivity of the vector declined drastically when the temperature was raised above 20 °C. Throughout a long-term stability study, formulations containing 10% sugar (sucrose/trehalose), 10% mannitol, 0.5% gelatin, and 10 mM histidine showed satisfactory stability for six months at 2–8 °C. The development of this freeze-drying process and the optimized formulation minimize the need for a costly cold chain distribution system.

## 1. Introduction

The recent SARS-CoV-2 outbreak has raised concerns about global health, prompting the development of vaccines to lower its severity and limit its spread. Multiple vaccine platforms, particularly viral vector and mRNA vaccines, have been developed, with excellent efficacy in response to this sudden outbreak [1]. Despite their rapid development, these vaccines were found to be unstable and require storage at ultra-low or sub-zero temperatures [2,3]. Therefore, a strictly monitored cold chain distribution system is required for transport and storage. This is particularly challenging in low- and medium-income countries with an intermittent or deficient power supply [4]. Insufficient cold chain systems in these countries hinder the effectiveness of vaccination programs and subsequently lead to a decrease in the potency of delivered vaccines [5]. The World Health Organization (WHO) approximates that over half of all vaccines worldwide might go to waste annually due to challenges in maintaining the cold chain during transportation and other related issues [6]. As a result, the overall cost of vaccines also increases. Therefore, evaluating the factors that contribute to vaccine loss is critical to the improvement of vaccine stability and to minimizing costs [5].

The notion of “vaccine instability” refers to the phenomenon whereby a vaccine gradually loses its potency over time. Several factors contribute to vaccine instability, including temperature fluctuations, pH changes, freeze–thaw stresses, chemical reactions, oxidation, and contamination. Among these factors, the temperature has a significant impact, particularly on live viral enveloped vector vaccines. Exposure to temperatures that are too high or too low can denature or damage the vaccine’s components, rendering the vaccine ineffective [7]. Furthermore, repeated freeze–thaw cycles can lead to the physical breakdown of the viral vector particles and the genetic material. Therefore, proper formulation and storage conditions, including control of the pH and osmolarity and the addition of stabilizers, are crucial in maintaining the stability of viral vaccines [8,9].

Most viruses are unstable in liquid form above refrigerated temperatures due to water-mediated destabilization and degradation mechanisms [9,10]. To overcome this instability, freeze-drying is used to dehydrate viral vector vaccines, immobilizing their structures and biological activity [11]. This technique is effective for the long-term storage of viral vector vaccines [12,13]. Several parameters of the freeze-drying cycle, known as critical process parameters (CPPs), can influence the quality of live viral vector vaccines. These CPPs include the freezing rate, ramp rate, temperature, pressure, pH, storage vials, buffers, and cryoprotectants [14,15]. These parameters must be evaluated and controlled carefully to minimize the loss of viral infectivity. Moreover, the final formulation of the freeze-dried vaccine must meet certain quality thresholds to be accepted as a successful solid formulation. These qualities are generally known as critical quality attributes (CQAs) and include thresholds describing the cake stability, resuspension efficiency, and moisture content [16,17].

**Figure 1:**
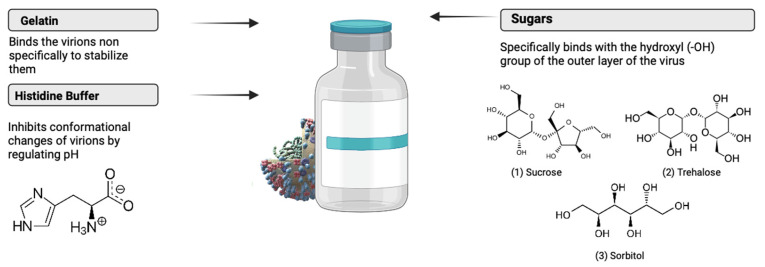
Mechanisms by which different excipients and buffers stabilize enveloped viral vectors [12,24,25]. Figure created with Biorender.com (accessed on 25 March 2024).

In contrast to non-enveloped viruses, enveloped viruses exhibit greater susceptibility to freeze-drying stress due to their fragile lipid bilayer [10]. To maintain their infectivity and functionality, they must preserve their envelope structure [12,16]. To minimize the stresses induced during freeze-drying, sufficient cryoprotection media are required during the process [18]. Sugars like sucrose and trehalose are often recommended as suitable stabilizers since they retain their amorphous structure throughout the process (Figure 1) [19]. In comparison to other monosaccharides, these sugars have a high glass transition temperature (T_g_) and high collapse temperature (T_c_) [17,20]. This increases the chemical potential of the vaccine and ensures the compact state of the structure [21]. Cryo-concentrated sugars improve the stability by reducing the reaction rates and inhibiting the structural mobility [12]. These sugars also confer both colloidal and conformational stability to the viral vector under extreme conditions [11]. Moreover, stabilizers like gelatin are used in freeze-dried live viral vector vaccines. Gelatin’s gelling properties are often utilized to protect vaccines against temperature-induced inactivation (Figure 1) [22].

In this study, we present the design and development of a freeze-drying process for the enveloped vector rVSV-SARS-CoV-2. VSV, as an enveloped virus, is considered a promising viral vector vaccine because it can elicit both strong humoral and cellular immune responses [7]. The WHO has classified many viruses as emerging infectious diseases, and VSV has been used in several initiatives as a vaccinology platform to combat these diseases. Research conducted over a period of more than ten years has shown that VSV has significant promise as a vehicle for the delivery of vaccines against several infectious diseases, including Ebola, Marburg, Lassa, Nipah, Zika, CCHF, MERS, and SARS. A VSV-based Ebola vaccine was recently approved for human use and another VSV-based COVID-19 vaccine is under development [23]. In both cases, the VSV envelope glycoprotein G was replaced by the Ebola virus glycoprotein or the SARS-CoV-2 spike antigen, resulting in a replication-competent virus. Further, vaccine candidates using VSV as a vector are under development for a range of illnesses, such as severe fever with thrombocytopenia syndrome; the West Nile, Chikungunya, and Andes viruses; and Dengue. Promising outcomes have been shown in animal experiments for the vector vaccines against Marburg virus, Nipah virus, Zika virus, and Lassa virus [7]. The advancement of rVSV vaccine candidates in human trials may aid in the development of efficacious vaccination strategies for outbreaks, contributing to pandemic preparedness.

A brief literature review was performed to determine the viral vector product formulations currently used in both the vaccine and gene therapy fields. Table 1 highlights some of the excipients that were found to be effective in freeze-dried viral vaccine products.

Here, several formulations were initially screened and those that could reduce the freeze-drying stresses were selected. CPPs like the temperature, pressure, time, and ramp rate were optimized at different stages of the freeze-drying process. Throughout this optimization, CQAs like cake formation, cake stability, resuspension efficiency, and moisture content were evaluated in the development of the freeze-drying process. The freeze-dried viruses from different process stages and their long-term stability were finally evaluated through an infectivity assay.

## 2. Materials and Methods

### 2.1. Cells and Viruses 

Suspension Vero cells (originated from ATCC CCL-81) were cultured in Kamen’s lab (McGill University) and used for this study [34]. They were grown and maintained in serum-free VP-SFM (Gibco, New York, NY, USA) medium supplemented with 4 mM GlutaMAX (Thermo Fisher Scientific, Waltham, MA, USA). The rVSV-SARS-CoV-2 construct was obtained from Kang’s laboratory in the Department of Biology, University of Western Ontario, Canada [35,36].

### 2.2. Production and Purification of Virus Samples

Vero cells were cultivated in shake flasks in a 25 mL working volume at 37 °C and subsequently increased to a 500 mL working volume. At the desired density (1–2 million cells/mL), the Vero cells were collected, pooled, and seeded at the same concentration (1–2 million cells/mL) in a fresh medium. Immediately after media transfer, the fresh Vero culture was infected with rVSV-SARS-CoV-2 at a multiplicity of infection (MOI) of 0.01 and the temperature was reset to 31 °C for optimal infection and production [36]. The harvest was collected after 48 h when the cell viability was less than 50%. The harvest was then treated with benzonase (Millipore Sigma, Burlington, MA, USA). Next, the virus was first centrifuged using a Megafuge 16R centrifuge (Thermo Fisher Scientific, Waltham, MA, USA) at 800× *g* for 5 min for cellular debris removal. After centrifugation, the supernatant was collected and the pellet containing cell debris was discarded. Then, the material was filtered using 0.45 µm syringe filters (Millipore Sigma, USA). The harvest was then run through an anion exchange chromatography machine, the Akta Avant (Cytiva, Marlborough, MA, USA), using elution buffer A (20 mM of Tris–HCl) and elution buffer B (20 mM of Tris–HCl and 2 M of salt) [37]. The mean virus titer was found as 6.61 × 10^9^ TCID_50_/mL and the standard deviation was 3.02 × 10^9^. The purified virus was stored at −80 °C.

### 2.3. Cryoprotectant, Lyoprotectant, and Buffer

Sorbitol and trehalose were acquired from Fisher Scientific, USA; sucrose and gelatin from Millipore-Sigma, USA; mannitol from Bio Basic, Toronto, Canada; and histidine from VWR, Radnor, USA. We used hydrolyzed gelatin for this freeze-drying study, with a molecular weight ranging between 40,000 and 50,000 Da, according to the supplier. To evaluate the stability of a formulation, 100 μL of the virus sample was mixed with 900 μL of a solution containing different concentrations of the cryoprotectant, lyoprotectant, and histidine buffer, as outlined in Table 2.

### 2.4. Freeze-Drying 

The freeze-drying of rVSV-SARS-CoV-2 was carried out using the Virtis Advantage Pro (SP Scientific, Warminster, PA, USA). This freeze dryer has recently been used for the lyophilization of an adenoviral vector against Newcastle Disease Virus [38]. For the freeze-drying study, 3 mL amber glass vials and butyl rubber stoppers from WHEATON^®^, Millville, NJ, USA were used. All vials were autoclaved, and all solutions were filtered through 0.22 μm capsule filters before freeze-drying. For freeze-drying, 1 mL of solution was prepared in 5 mL glass vials containing the virus and the excipients. After the completion of each cycle, all vials were sealed, and no inert gas was used in the vacuum process. In the first step of the freeze-drying cycle, freezing was performed at −50 °C for five hours. Primary drying was conducted for 15 h at −30 °C. During the secondary drying, the temperature was gradually increased to remove the residual water. Following the achievement of the desired temperature, all formulations were kept for a particular drying time to reach their required moisture levels. To evaluate the freeze-drying stresses, the loss of infectivity of rVSV-SARS-CoV-2 was determined by a viral infectivity assay_._

### 2.5. Stability Study

At the specified time points (0 days, one week, one month, six months), the solid, freeze-dried cakes were removed from their designated storage temperatures (4, 20, and 37 °C) and resuspended in a phosphate-buffered saline (PBS) solution. The infectious viral titer was then determined using the infectivity assay described in Section 2.6. 

### 2.6. Viral Infectivity Assay

The infectivity dose of the virus was determined using a cell culture-based median tissue culture infectious dose (TCID_50_) assay. For the quantification of the viral titer, 100 μL of cell culture media containing 15,000 adherent Vero cells was seeded in each well of a 96-well plate. One 96-well plate was used for each lyophilized formulation and these plates were kept at 37 °C with 5% CO_2_. After 24 h, the medium was removed and replaced by fresh medium containing a serial dilution of the formulation (1:10). Next, the temperature was set to 31 °C with 5% CO_2_ and kept for 4 days. After 4 days of incubation, the wells were analyzed using an inverted microscope to determine the cytopathic effect (CPE), which is characterized by rounded cells, a disrupted monolayer, and/or clumping. The infectious viral titer was calculated by the Spearman and Kärber algorithm by determining the number of positive complete CPE wells in each column (mean coefficient of variation: 30.85%; Supplementary Figure S1) [37,39].

### 2.7. Moisture Content Determination

Moisture content was determined using a laboratory scale, R3005 (Sartorius, Göttingen, Germany). The weight of the excipients was known, and the weight of the empty vials was determined. After filling 1 mL of formulation into each glass vial, the weight was measured before and after the freeze-drying cycle. The extra weight was calculated as water by subtracting the known mass of the excipients [38,40].

### 2.8. Statistical Analysis

For the statistical analysis, one-way analysis of variance (ANOVA) tests were performed using the GraphPad Prism application. The data from this study followed a Gaussian distribution, as visually confirmed by QQ plots. Dunnett’s multiple comparisons tests were performed between the control (unformulated) and the formulated groups with a 95% confidence interval of difference. Statistically significant differences between the formulation groups were defined at a *p*-value of less than 0.05, and these statistically significant differences were presented through asterisks (* sign). Moreover, the JMP application and the Microsoft Excel application were used to compare the data from different formulation groups.

## 3. Results

### 3.1. Effect of Final Moisture Content

**Figure 2:**
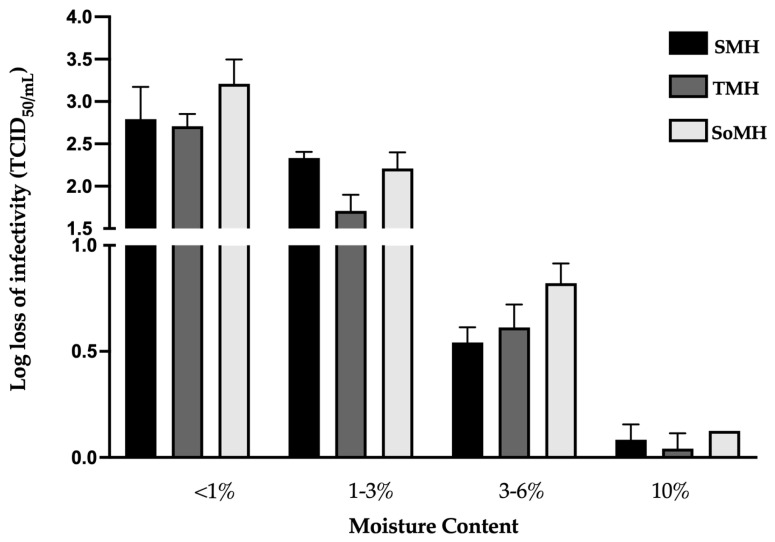
Effect of final moisture content on infectivity of freeze-dried rVSV-SARS-CoV-2. Here, the infectious viral titer is presented as the log loss of infectivity. The log loss was estimated by deducting the log(titer) of a controlled −80 °C stock sample from the log(titer) of the formulation sample. The infectious viral titer is the average of three samples’ TCID_50_ results; error bars represent the standard deviation. The moisture content was measured by the weight loss method in each cycle. The mean value and SD of the moisture measurements are shown in Table S1 in the Supplementary Materials.

This part of the research aimed to determine the ideal moisture range, which is considered one of the CQAs for the enveloped rVSV-SARS-CoV-2 vector. Three primary formulations (SMH, TMH, and SoMH) were selected (Table 2) and evaluated at four different moisture ranges (<1%, 1–3%, 3–6%, and 10%). This study revealed that extreme moisture content levels ultimately led to poor stability or cake deterioration (<1% or 10%). Furthermore, we found that a narrow range of final moisture content led to the comparatively high recovery of the infectious viral titer (3–6%) and enhanced the cake stability. Figure 2 displays the TCID_50_ results of infectious viruses lyophilized to various moisture content levels.

There was a clear trend indicating that the infectious viral titer recovery increased as the final moisture content increased. The highest loss of infectious viral titer was observed at <1% moisture content, showing a ~2.5 to 3.5 log loss of infectivity across all three formulations. Within the moisture range of 1–3%, the change in infectious viral titer varied between the three formulations. The trehalose-containing formulation (TMH) showed a 1.7 ± 0.2 log loss of infectious viral titer, whereas both the sucrose- (SMH) and sorbitol-containing (SoMH) formulations showed >2. Within the final moisture content range of 3–6%, all formulations demonstrated an infectious viral log loss of ~0.5 to 0.7. Finally, at 10% final moisture content, despite no loss of infectious viral titer being observed, the lyophilized cake collapsed within 24 h. 

These findings emphasize the presence of a specific final moisture range in preserving rVSV-SARS-CoV-2’s infectivity during the freeze-drying process. The optimal moisture level (3–6%) resulted in high viral titer recovery and stable cake formation, indicating the suitability of this range for lyophilized rVSV-SARS-CoV-2 formulations. These results emphasize the need for the precise control of the moisture content to ensure that freeze-dried viral products maintain high efficacy and long shelf lives.

### 3.2. Effect of Formulation’s Composition and Concentration

**Figure 3:**
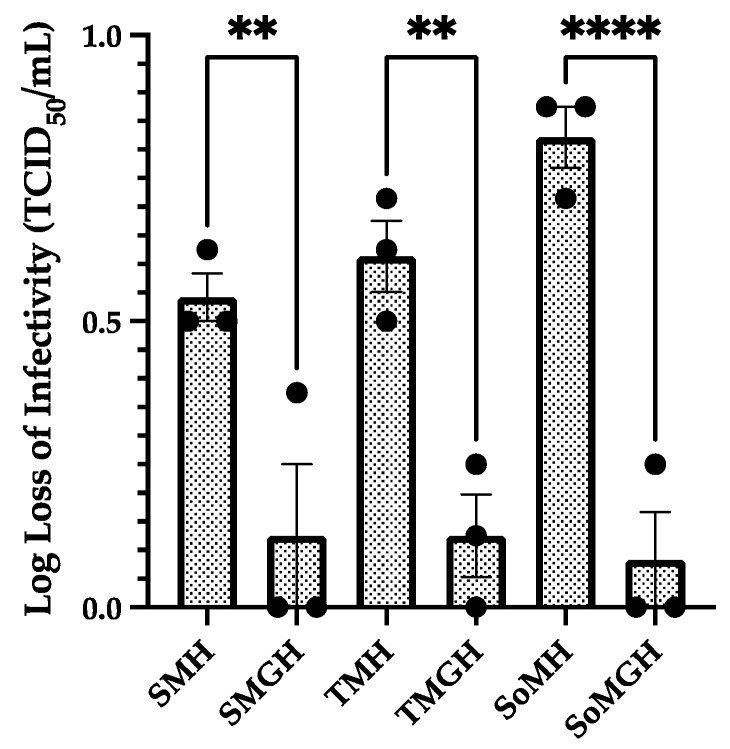
Effect of formulations’ concentrations and compositions on infectivity of rVSV-SARS-CoV-2. Six different formulations of rVSV-SARS-CoV-2 were freeze-dried and the infectivity of each was determined by the TCID_50_ assay. Here, the infectious titer of rVSV-SARS-CoV-2 is presented as the log loss of infectivity calculated by deducting the log(titer) of a controlled −80 °C stock sample from the log(titer) of the formulation sample. The values shown are the average of three samples’ TCID_50_ log loss results, and the error bars represent the standard deviation. One-way ANOVA analysis was performed between specific formulation groups, and the significant statistical difference is denoted through the asterisks (*) sign. Significant statistical differences were identified when the *p*-values were less than 0.05 (**** *p* < 0.0001; ** *p* < 0.01).

Next, as a stepwise improvement in the freeze-dried formulations, the compositions and concentrations of the excipients were evaluated. Here, the optimized moisture content (~3–6%) from Section 3.1 was considered. We also ensured proper cake formation and cake stability for all samples in this part of the study. In this part of the analysis, we observed that the excipient concentrations had a major impact on the infectivity of the freeze-dried formulations. Initially, formulations predominantly composed of 5% cryoprotectant and 5% lyoprotectant exhibited a notable loss in infectious viral titer (~0.5 to 0.8 log loss) after freeze-drying. To investigate this phenomenon, we systematically increased the concentrations of both the cryoprotectant and lyoprotectant from 5 to 10%, and 0.5% gelatin was added. These were labeled SMGH, TMGH, and SoMGH, as given in Table 2. These modified formulations demonstrated a remarkable improvement in the recovery of infectivity. Notably, all new formulations showed a <0.2 log loss of infectious viral titer. Figure 3 compares these new formulations with the previous formulations.

These results highlight the critical role of a formulation’s composition and concentration in safeguarding viral integrity during freeze-drying processes. By increasing the concentrations of the cryoprotectants, and lyoprotectants and incorporating gelatin, these formulations effectively mitigated the detrimental effects of freeze-drying stress on the infectivity of rVSV-SARS-CoV-2. Moreover, the increased concentration of the lyoprotectant positively influenced the cake thickness, indicating improved structural integrity.

### 3.3. Effect of Temperature

To investigate the effect of the CPPs, we also studied the impact of several freeze-drying cycle parameters on the infectivity of rVSV-SARS-CoV-2. Among all of the CPPs evaluated, the temperature had the most significant effect on the infectivity of the viral vector and the structural integrity of the lyophilized cake. Three optimized formulations (SMGH, TMGH, and SoMGH) were used to evaluate the temperature effect across all freeze-drying stages, while considering the previously optimized moisture content range and the cake stability.

In the freezing stage, samples were taken at the middle and end of the process. No decline in the infectious virus titer was found throughout the freezing phase. Different temperature ramp rates, from 0.1 to 1 °C/min, were tested in the freezing stage to investigate the potential influence on the infectivity of the viral vector. These variations in ramp rate showed no influence on the infectivity. 

In the next phase, the temperature of the primary drying was set to −30 °C based on the T_g_ and T_c_ values of the formulations. The primary drying stage was the longest step of the freeze-drying cycle, taking approximately 15 h. During this step, no loss of infectious viral titer was observed. In addition, different ramp rates (0.1 to 1 °C/min) used in the primary drying showed no loss of viral titer.

**Figure 4:**
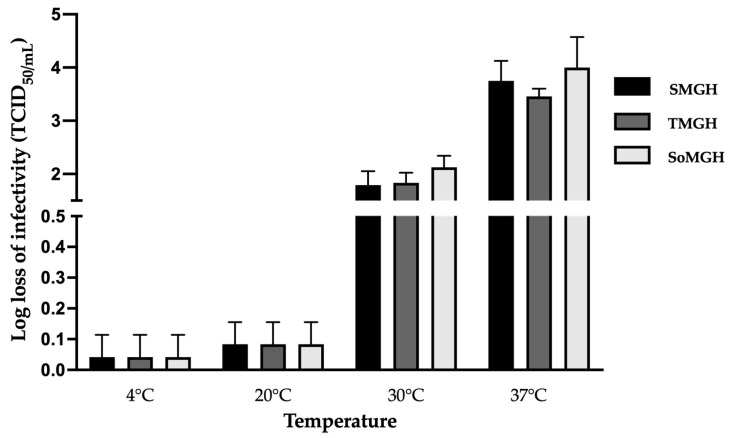
Effect of secondary drying temperatures on three formulations (SMGH, TMGH, and SoMGH) containing rVSV-SARS-CoV-2. Four different temperatures—4, 20, 30, and 37 °C—were applied to the secondary drying step of the freeze-drying cycle. The log loss of rVSV-SARS-CoV-2 was calculated by deducting the log (titer) of a controlled −80 °C stock sample from the log (titer) of the formulation sample. The values shown are the average of three samples’ TCID_50_ log loss results, and the error bars represent the standard deviation.

**Figure 5:**
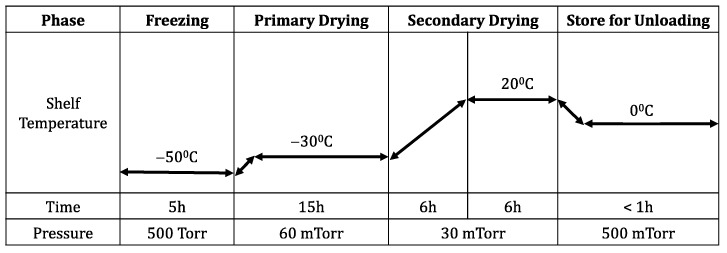
An optimized freeze-drying cycle was developed for rVSV-SARS-CoV-2. This freeze-drying cycle was designed for 1 mL samples comprising optimal SMGH, TMGH, and SoMGH formulations. Freezing was performed at −50 °C for 5 h with no vacuum. Next, primary drying was performed for 15 h at −30 °C based on the T_g_ and T_c_ of the formulations. A fast ramp rate of ~1 °C/min was applied both in the freezing and primary drying stages, whereas a slow ramp rate of ~0.1 °C/min was applied in the secondary drying stage. Lastly, the secondary drying was performed at 20 °C for 6 h.

During the secondary drying step, the temperature is gradually increased, and the vacuum pressure is lowered to evaporate the residual water. These extreme conditions induce more stress on the viral vector. To assess the impact of the temperature during this phase, several temperatures were tested. Secondary drying at refrigerated temperatures (4 °C) brought no significant loss of viral titer (<0.1 log loss of infectivity). To reduce the drying time, the secondary drying temperature was increased from 4 to 20 °C, where the three formulations also showed no major loss (~0.1 to 0.2 log loss) of infectious viral titer (Figure 4). However, when the temperature of the secondary drying was raised above 20 °C, a significant loss of infectivity in the vector was observed. In the three formulations, secondary drying at 30 and 37 °C showed a 2 ± 0.5 and 3.8 ± 0.7 log loss of infectious viral titer, respectively. The observed infective viral titer loss in all three formulations indicates that the excipients used in these formulations were not successful in preventing viral loss at elevated temperatures. Based on these findings, the secondary drying temperature was set to 20 °C to reduce the significant losses and minimize the duration of this process (Figure 4). Here, a slow ramp rate of ~0.1 °C/min was applied from primary drying to secondary drying to minimize the freeze-drying stress (Figure 5).

Lastly, we observed the reconstitution stability of the three optimized formulations (SMGH, TMGH, and SoMGH) for 24h at 4 °C and found no loss in the infectious viral titer (Supplementary Figure S2).

### 3.4. Long-Term Stability Data

**Figure 6:**
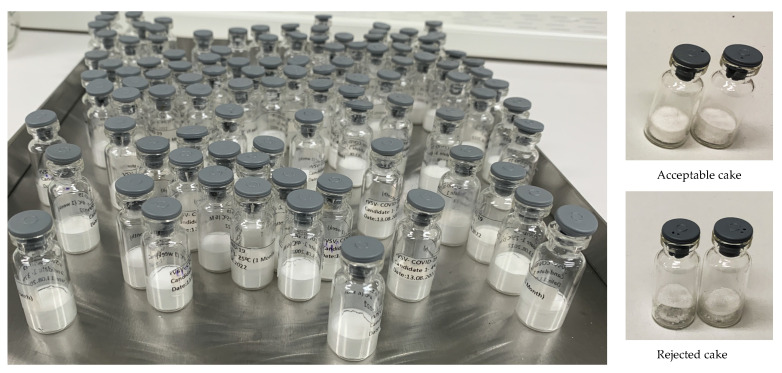
A small batch of lyophilized rVSV-SARS-CoV-2 cakes using formulations of SMGH, TMGH, and SoMGH is shown. In most cases, the cake appearance is considered a quality attribute and must meet certain criteria for acceptance. In the final stage depicted in the figure, the lyophilized cake is fully formed, presenting a uniform and porous structure. After optimizing the CPPs and CQAs, each vial in the figure shows perfect cake formation without any cake deterioration or shrinkage. The cake adheres firmly to the vial walls, indicative of successful lyophilization and optimal cake stability. Notably, the absence of collapse or cracking underscores the efficacy of the process in maintaining the structural integrity of the cake.

Following the optimization of the formulations and critical parameters of the freeze-drying cycle, we manufactured a small batch of freeze-dried vaccine vials (Figure 6). To investigate the thermal stability, these optimized freeze-dried formulations, SMGH, TMGH, and SoMGH, were stored at 4, 20, and 37 °C for up to six months. The unformulated samples (control) were incubated at the same conditions to compare the stability results.

**Figure 7:**
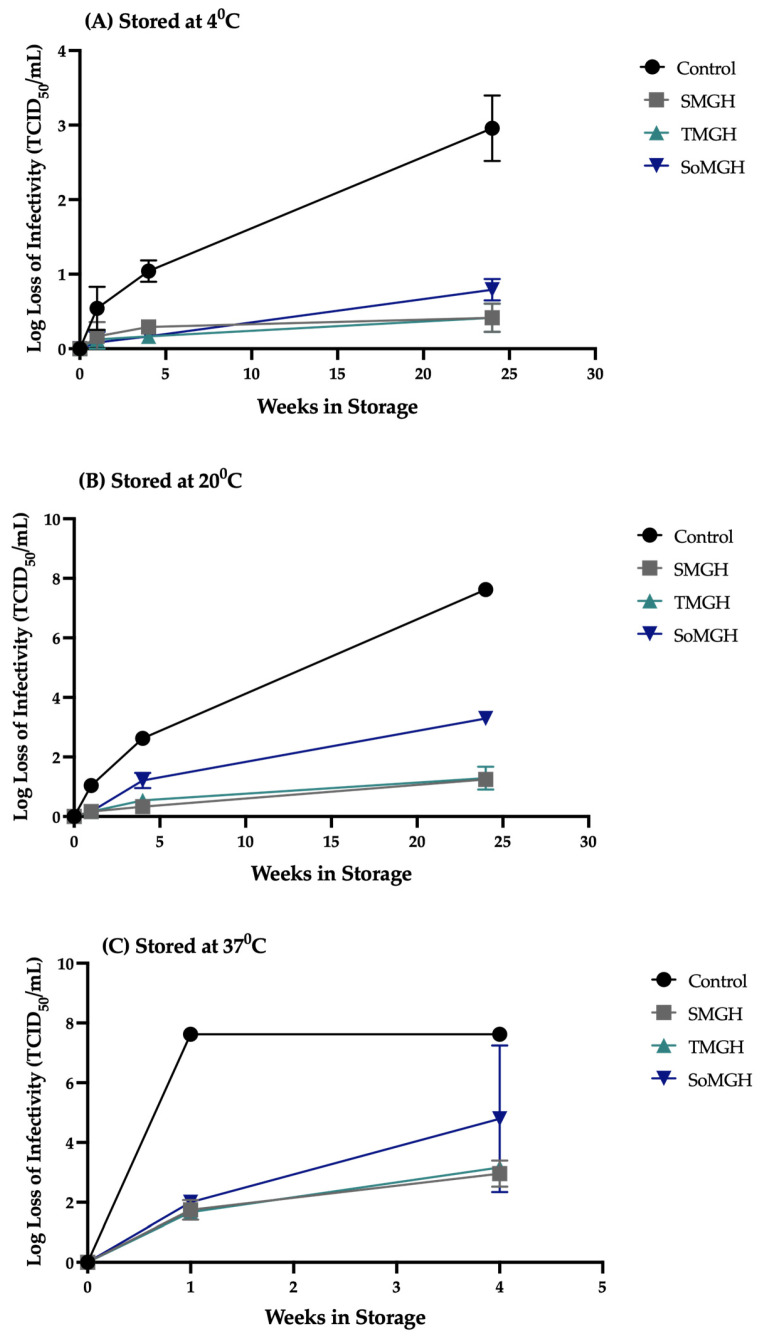
The loss of infective viral titer of three distinct rVSV-SARS-CoV-2 formulations that were observed over time at temperatures of 4 °C (**A**), 20 °C (**B**), and 37 °C (**C**) is shown. The TCID_50_ viral assay was performed for three formulation groups and one control (unformulated liquid sample) to detect the infectious viral titer. The viral titer is presented as a log loss value on the y-axis. Log losses of the viral titer due to thermal stress were calculated in comparison to initial control samples (−80 °C stock); data are displayed as the mean ± standard deviation (*n* = 3).

After one week at 37 °C, the control showed the complete loss of the infectious viral titer (Figure 7C). In contrast, the titer losses were reduced by the three freeze-dried formulations in this accelerated stress condition (37 °C). More specifically, SoMGH (sor-man-gel-his) showed 2 ± 0.2 log loss, whereas SMGH (suc-man-gel-his) and TMGH (tre-man-gel-his) showed 1.75 ± 0.25 log loss (Figure 7A). From the ANOVA, statistically significant differences were found between the control and the other three formulations at 37 °C (Supplementary Figure S3a). This indicates that the optimized formulations played a vital role in minimizing the effect of the temperature stress on the infectivity of rVSV-SARS-CoV-2.

In contrast, the three freeze-dried formulations showed only a small loss of infectious viral titer at 4 and 20 °C after one week. At 20 °C, the three formulations showed a <0.3 log loss of infectivity, while the control showed a ~1 log loss of infectivity (Figure 7B). At 4 °C, on the other hand, the three formulations showed a ~0.1 to 0.2 log loss, with the control having a 0.5 ± 0.2 log loss (Figure 7A). This demonstrates that the three formulations had improved stability when exposed to 4 and 20 °C for a short period. There were no statistically significant differences (*p* > 0.05) found between the control and other groups at 4 °C (Supplementary Figure S3c). However, a statistically significant difference (*p* < 0.0001) was identified at 20 °C between the control and the other three formulations (Supplementary Figure S3b). This indicates that these formulations have significant value in protecting the viral vector in this condition.

The stability study was extended to one month under the same conditions as the one-week test. As shown in Figure 7C, a significant loss of infectious viral titer was observed for rVSV-SARS-CoV-2 in the accelerated (37 °C, 1 month) stress condition. Here, the sorbitol-containing formulation (SoMGH) showed a severe loss of infectivity close to the control, and the other two formulations (SMGH and TMGH) showed a ~3 log loss of infectivity (Figure 7C). More importantly, there were less statistically significant differences found between the control and SMGH (*p* = 0.0079) and between the control and TMGH (*p* = 0.0102) (Supplementary Figure S4a). However, no statistically significant difference was found between the control and SoMGH (*p* = 0.0913), suggesting that the formulation incorporating sorbitol had poor stability in this condition. 

After one month at 20 °C, distinct stability data were observed among the three formulations. Figure 7B shows a significant loss (1.25 ± 0.25 log loss) of viral titer in the SoMGH formulation. Unlike SoMGH, SMGH showed a 0.3 ± 0.1 and TMGH showed a 0.5 ± 0.1 log loss of infectivity. Therefore, a less statistically significant difference was found between the control and SoMGH as compared to the other two formulations (Supplementary Figure S4b). Following one month at 4 °C, the measured infectious viral titer was found to be high in all formulations (0.1 to 0.3 log loss of infectivity). Under this condition, a statistically significant difference (*p* < 0.0001) was observed between the control and all other formulation groups, as the control showed a ~1 log loss of infectivity (Supplementary Figure S4c). The statistical analyses demonstrate that the excipients effectively shielded the viral vector from denaturation, aggregation, and loss of infectivity at both 4 and 20 °C temperatures.

The complete loss of the infectious vector rVSV-SARS-CoV-2’s titer was observed in all formulations when the stability study was carried out for six months at 37 °C (no figure shown). Among the three formulations tested, the sucrose-containing SMGH and trehalose-containing TMGH formulations were found to be effective in maintaining the viral titer for six months at 4 °C (<0.5 log loss of infectivity, Figure 7C). Further, both of these formulations also showed promising stability for 6 months at 20 °C (~1.2 log loss of infectivity, Figure 7B). In contrast, the sorbitol-containing formulation SoMGH showed a significant drop in infectious viral titer within six months both at 4 and 20 °C (Figure 7A,B). Here, it appears that sorbitol as a cryoprotectant was unsuccessful in stabilizing rVSV-SARS-CoV-2 as compared to the other two formulations containing sucrose and trehalose. The statistically significant difference observed between the control and the two improved formulations (SMGH and TMGH) in 6 months at both 4 and 20 °C demonstrates their ability to improve the shelf life of the viral vector.

## 4. Discussion

To achieve a successful solid formulation of a viral vector vaccine, two challenges must be overcome. The first is to protect the vector from the in-process stresses of the freeze-drying cycle; the second is to protect the vector during long-term storage and transportation [41]. The primary objective of this freeze-drying study was to maintain a high virus titer. The stability analysis showed that sucrose–gelatin, trehalose–gelatin, and sorbitol–gelatin formulations play an important role in maintaining the rVSV-SARS-CoV-2 during the freeze-drying cycle. These formulations create a protective environment that effectively mitigates the adverse effects of freezing and dehydration. Furthermore, the virus titer was found to be high in the sucrose–gelatin and trehalose–gelatin formulations when their long-term stability was examined. Therefore, these two freeze-dried formulations may be considered for potential use for this COVID-19 vaccine. 

The type and concentration of the cryoprotectant, lyoprotectant, and buffer also played a major role in maintaining the infectivity of VSV. The freeze-drying stress on enveloped viruses is caused by the fast expansion of small ice crystals during warming in the secondary drying phase. This increased rate of ice recrystallization compromises the structure of the envelope, resulting in the significant loss of the virus [26,42]. Under this stress condition, the maximum recovery of the viral titer can be obtained by increasing the concentrations of the sugar excipients [22]. Similarly, this freeze-drying work showed that increasing concentrations of sugar and the presence of gelatin played a vital role in the infectivity of rVSV-SARS-CoV-2. The high concentrations of sugar excipients and the gelatin contributed significantly to retaining the infectious viral titer throughout the freeze-drying process. In the case of Herpes Simplex Virus, which is classified as a heat-labile enveloped virus, high concentrations of cryoprotectant (27% *w*/*v*) were shown to recover almost 80% of the viral titer [43,44,45,46].

One objective of this study was to explore the impact of the moisture content on freeze-drying formulations. The purpose of the freeze-drying cycle is to remove most of the water from the formulation, but a minimum level of water activity is required to retain viral infectivity. In the case of viral vectors, it was found that overdried freeze-dried formulations had lower viral titers than formulations with slightly higher moisture content (1–3%) [47]. Based on our findings, overdried rVSV-SARS-CoV-2 also resulted in a major loss of infectivity. In contrast, higher moisture content contributed positively to the recovery of the viral vector, with the most optimized recovery found at 3–6%. As a comparison, the ideal final moisture content for influenza virus was determined to be 1.7% and drying them below this point made them non-infectious. Our results demonstrate that the rVSV-SARS-CoV-2 vector requires higher moisture content as compared to other viral vectors to retain its infectivity.

The temperature is known to be a critical factor in the freeze-drying process that affects the stability of viruses. In the primary drying phase, the temperature needs to be adjusted below the collapse temperature (T_c_). If this temperature is exceeded, the frozen product structure will collapse [47]. This may lead to high moisture content and an unsatisfactory product appearance. Therefore, the primary drying temperature must be optimized for product quality and a robust primary drying rate [48]. The primary drying process for all three formulations was conducted at −30 °C based on the T_c_. At this temperature, neither the product quality nor the infectivity declined during primary drying.

Following the primary drying phase, secondary drying is carried out to evaporate the bound water. Usually, secondary drying is performed at high temperatures as more energy is required for the evaporation [49]. The secondary drying process requires a gradual increase in the shelf temperature or a microcollapse may occur [48]. Furthermore, rapid temperature changes during the drying process may lead to the denaturation or inactivation of the virus. For this reason, it is vital to maintain appropriate temperatures during secondary drying to protect the virus’ infectivity. We aimed to develop a robust process in this study with an optimal secondary drying temperature. This study found that there is a critical temperature range (≤20 °C) within which the infectious viral titer remains stable during secondary drying. However, once the temperature surpasses this range, there is a notable decrease in the infectious viral titer.

Additionally, pH changes were recorded for all formulations and slight changes in pH were observed in all formulations before and after freeze-drying (Supplementary Table S2). Although this work did not investigate the impact of pH variations on VSV infectivity, future elaborations on this study can include a pH study to determine its effect on infectivity.

To minimize the infectious viral titer loss, the entire freeze-drying cycle is normally carried out at ultra-low temperatures in many live viral vector vaccines. In the case of live attenuated viruses, the total time for the freeze-drying cycle normally exceeds 50 h [12]. However, from an economical and industrial standpoint, these lengthy freeze-drying cycles are costly and time-consuming. This study developed a freeze-drying process that took only 32 h to complete and is considered a robust process with no significant loss of infectivity. This robust process ensures that the final product meets the aforementioned quality specifications consistently over multiple production runs. It can also reduce the operating costs associated with energy consumption and labor, making it cost-effective for large-scale industrial production.

For this study, the selection of the TCID_50_ was considered because this assay is a well-established and standardized method in determining the viral titer. The assay can detect low levels of infectious viral titer. When properly conducted, it can yield consistent and reproducible results, which is crucial for this set of experiments. The assay is inexpensive and easy to implement, especially when virus-specific antibodies are not available [39]. As VSV demonstrates a clear cytopathic effect on adherent Vero cells and it is easy to distinguish between the infected and non-infected cells, the TCID_50_ has been considered as a primary indicator of function and the critical quality attribute of the viral product for this study. 

The improved stability of VSV developed from this freeze-drying analysis may potentially address the stability limitations in many vaccine candidates. For example, to ensure stability, the recently approved Ebola vaccine (Ervebo, a VSV-based vaccine) requires storage between −80 and −60 °C. Once thawed, the vaccine can be stored at 2 to 8 °C for up to 14 days [50]. This study’s findings can alleviate the burden of the costly cold chain systems required. Moreover, this research makes a valuable contribution to the broader understanding of VSV’s stability through a meticulous assessment of many of the critical variables associated with freeze-drying.

In conclusion, this paper outlines a stepwise improvement in VSV stability throughout the freeze-drying process, considering both CPPs (temperature) and CQAs (cake stability, resuspension efficiency, and moisture content). The formulations and process design that were developed throughout this study in the freeze-drying phase and long-term stability assessment have the potential to offer valuable insights into the overall stability of enveloped viral vector vaccines.

## Figures and Tables

**Table 1:** Freeze-dried viral vaccine formulations.

Virus	Type	Composition	Ref
Vaccinia Virus (Smallpox Vaccine)	DNA viruses. Enveloped	Mannitol, human serum albumin	[12]
Yellow Fever Virus	RNA viruses. Enveloped	Sorbitol, gelatin	[12]
Varicella-Zoster Virus	DNA viruses. Enveloped	Sucrose, gelatin, glutamate	[12]
Influenza Virus	RNA viruses. Enveloped	Trehalose, sucrose, and soy peptone	[26]
Measles Virus	RNA viruses. Enveloped	Sorbitol–gelatin	[27]
Polio Virus	RNA viruses. Non-enveloped	Urea	[28]
Pseudorabies Virus	DNA virus. Enveloped	Glutamate, sucrose, dextran, phosphates	[29]
Herpes Simplex Virus 2 (HSV-2)	DNA virus. Enveloped	Sucrose	[30]
Herpesvirus	DNA virus. Enveloped	Sucrose, monopotassium phosphate, dipotassium phosphate, monosodium glutamate, and bovine albumin powder	[31]
Rubella Virus	RNA viruses. Enveloped	Calcium gluconate–lactobionate	[32]
Foot-and-Mouth Disease Virus (FMDV)	RNA viruses. Non-enveloped	Inositol, sodium glutamate, and calcium lactobionate	[33]

**Table 2:** Six formulation candidates for the freeze-drying study of rVSV-SARS-CoV-2.

SMH	TMH	SoMH	SMGH	TMGH	SoMGH
Sucrose 5% Mannitol 5% Histidine 10 mM	Trehalose 5% Mannitol 5% Histidine 10 mM	Sorbitol 5% Mannitol 5% Histidine 10 mM	Sucrose 10% Mannitol 10% Gelatin 0.5% Histidine 10 mM	Trehalose 10% Mannitol 10% Gelatin 0.5% Histidine 10 mM	Sorbitol 10% Mannitol 10% Gelatin 0.5% Histidine 10 mM

## Data Availability

The original contributions presented in the study are included in the article; further inquiries can be directed to the corresponding author.

## Supplementary Materials

The following supporting information can be downloaded at: https://www.mdpi.com/article/10.3390/v16060942/s1, Figure S1: Assay variability of TCID_50_ was assessed on same sample using three replicates on 0 day and after 3 months; Table S1. Moisture content range for Figure 2 (n = 6); Figure S2: Reconstitution stability of three formulations (SMGH, TMGH and SoMGH); Figure S3. After one week, the stability of three distinct rVSV-SARS-CoV-2 formulations at temperatures of 37 °C (a), 20 °C (b), and 4 °C (c) was assessed; Figure S4. After one month, the stability of three distinct rVSV-SARS-CoV-2 formulations at temperatures of 37 °C (a), 20 °C (b), and 4 °C (c) was assessed; Figure S5. After six months, the stability of three distinct rVSV-SARS-CoV-2 formulations at temperatures of 20 °C (a) and 4 °C (b) was assessed; Figure S6: Related to Figure 2 where one-way ANOVA analysis was performed between different moisture content groups of three formulations SMH, TMH and SoMH; Figure S7: Related to Figure 4 where one-way ANOVA analysis was performed between different temperature groups of three formulations SMGH, TMGH and SoMGH; Table S2: pH study of six different formulations before and after freeze-drying.
