# Supplementary material for: Development of Robust Freeze-Drying Process for Long-Term Stability of rVSV-SARS-CoV-2 Vaccine
Source: Viruses. 2024 Jun 11;16(6):942. doi: 10.3390/v16060942 (PMC11209311; doi:10.3390/v16060942)
Supplement: Supplementary file 1 [file viruses-16-00942-s001.zip › viruses-2964951-supplementary.pdf]

Supplementary Material

A

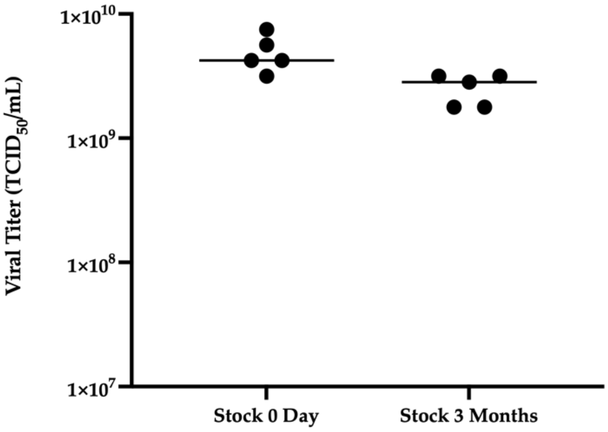

B

	0 Months	Stored 3 Months
**Mean**	4.944000000e+009	2.540000000e+009
**SD**	1.674897012e+009	7.075309181e+008
**Coefficient of Variability (%)**	33.877366740	27.855547950

Supplementary Figure S1. Assay variability of $TCID_{50}$ was determined by assaying the same sample using three replicates on 0 day and after 3 months.The mean coefficient of variability between both conditions was determined to be 30.8%.

Supplementary Table S1. Moisture content range for Figure 2 (n=6).

Label	Moisture Range
**< 1%**	0.42 ± 0.37 %
**1-3 %**	1.845 ± 0.53 %
**3-6 %**	4.23 ± 0.97 %
**10 %**	10.5 ± 0.36 %

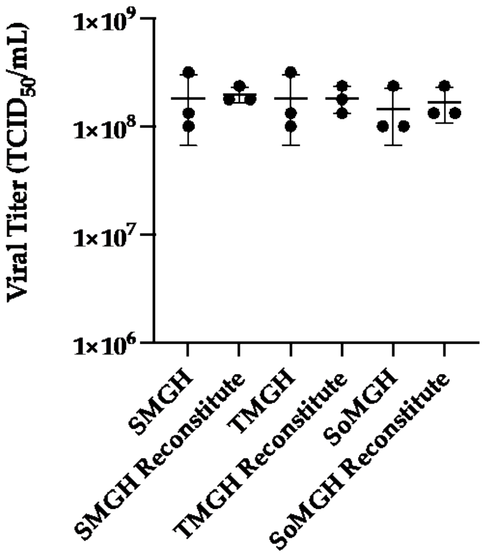

Supplementary Figure S2. Reconstitution stability of three formulations (SMGH, TMGH and SoMGH) were performed for 24 hours at 4 °C and compared with the immediate reconstituted same three formulations. Viral titer were detected by $TCID_{50}$ infectivity assay. Here phosphate buffer saline (PBS) was used a reconstitute buffer.

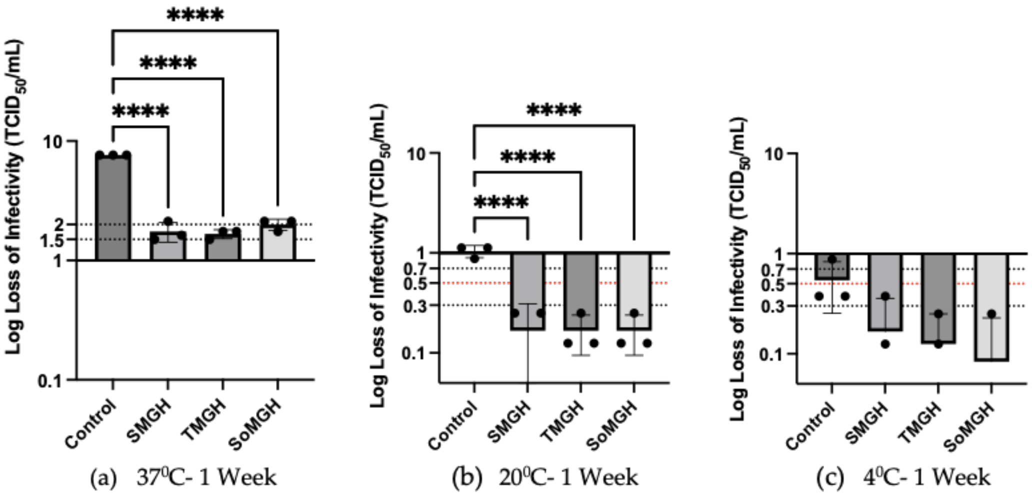

Supplementary Figure S3**.** After one week, the stability of three distinct rVSV-SARS-CoV-2 formulations at temperatures of 37 °C (a), 20 °C (b), and 4 °C (c) is shown. $TCID_{50}$ viral assay was performed for three formulation groups and one control to detect infectious viral titer. Viral titer is presented as a log loss value on the y-axis. Here, less than 0.5 log loss indicates good stability, and this boundary line is marked red. Log losses of viral titer due to thermal stress were calculated in comparison to control samples (−80 °C stock); data is displayed as the mean ± standard deviation ($n = 3$). One-way ANOVA analysis was performed between the control and formulation groups, and the significant statistical difference is denoted through the asterisks (*) sign. Significant statistical differences were identified when the p-values were less than 0.05 (****$p < 0.0001$; *** $p < 0.001$; ** $p < 0.01$; * $p < 0.05$).

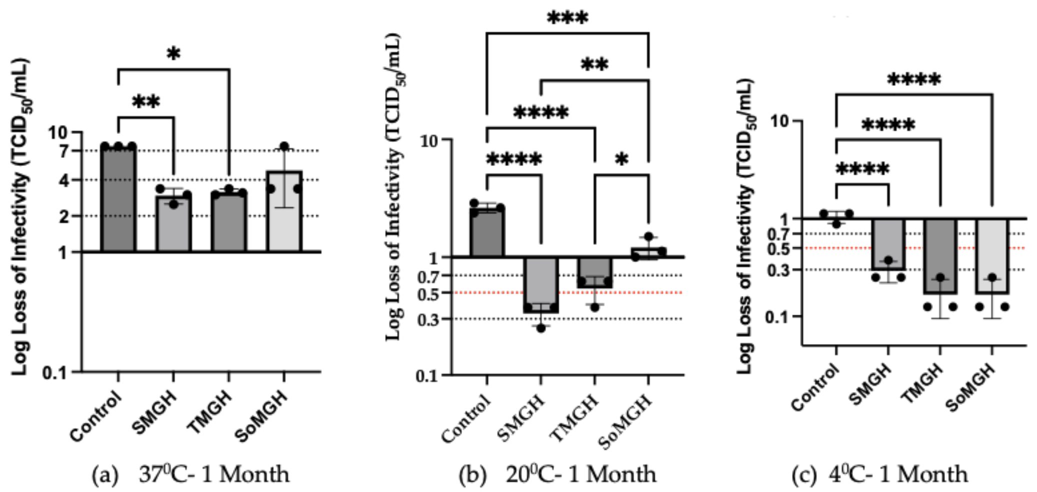

Supplementary Figure S4**.** After one month, the stability of three distinct rVSV-SARS-CoV-2 formulations at temperatures of 37 °C (a), 20 °C (b), and 4 °C (c) is shown. $TCID_{50}$ viral assay was performed for three formulation groups and one control to detect infectious viral titers. Viral titer is presented as a log loss value on the y-axis. Here, less than 0.5 log loss indicates good stability in the long-term storage and this boundary line is marked red. Log losses of viral titer due to thermal stress were calculated in comparison to control samples (−80 °C stock); data is displayed as the mean ± standard deviation (n = 3). One-way ANOVA analysis was performed between the control and formulation groups, and the significant statistical difference is denoted through the asterisks (*) sign. Significant statistical differences were identified when the p-values were less than 0.05 (****$p < 0.0001$; *** $p < 0.001$; ** $p < 0.01$; * $p < 0.05$).

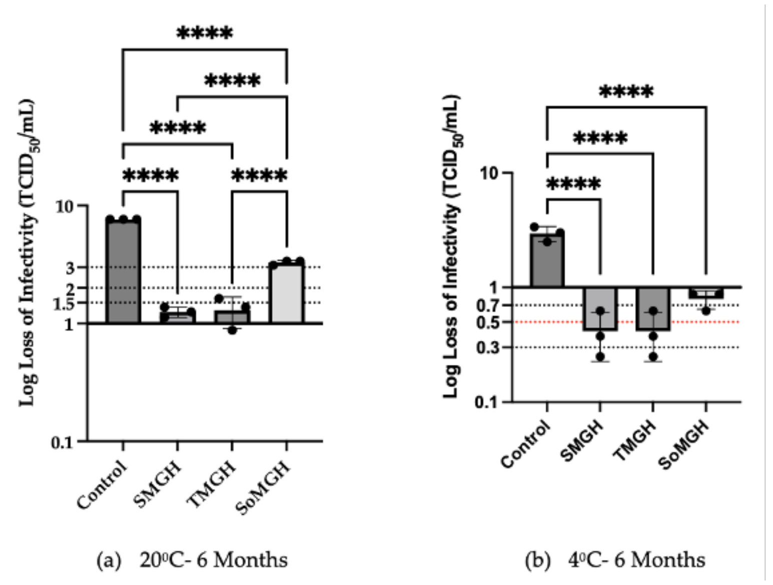

Supplementary Figure S5**.** After six months, the stability of three distinct rVSV-SARS-CoV-2 formulations at temperatures of 20 °C (a) and 4 °C (b) is shown. $TCID_{50}$ viral assay was performed for three formulation groups and one control to detect infectious viral titers. Viral titer is presented as a log loss value on the y-axis. Here, less than 0.5 log loss indicates good stability in the long-term storage and this boundary line is marked red. Log losses of viral titer due to thermal stress were calculated in comparison to control samples (−80 °C stock); data is displayed as the mean ± standard deviation (n = 3). One-way ANOVA analysis was performed between the control and formulation groups, and the significant statistical difference is denoted through the asterisks (*) sign. Significant statistical differences were identified when the p-values were less than 0.05 (****$p < 0.0001$; *** $p < 0.001$; ** $p < 0.01$; * $p < 0.05$).

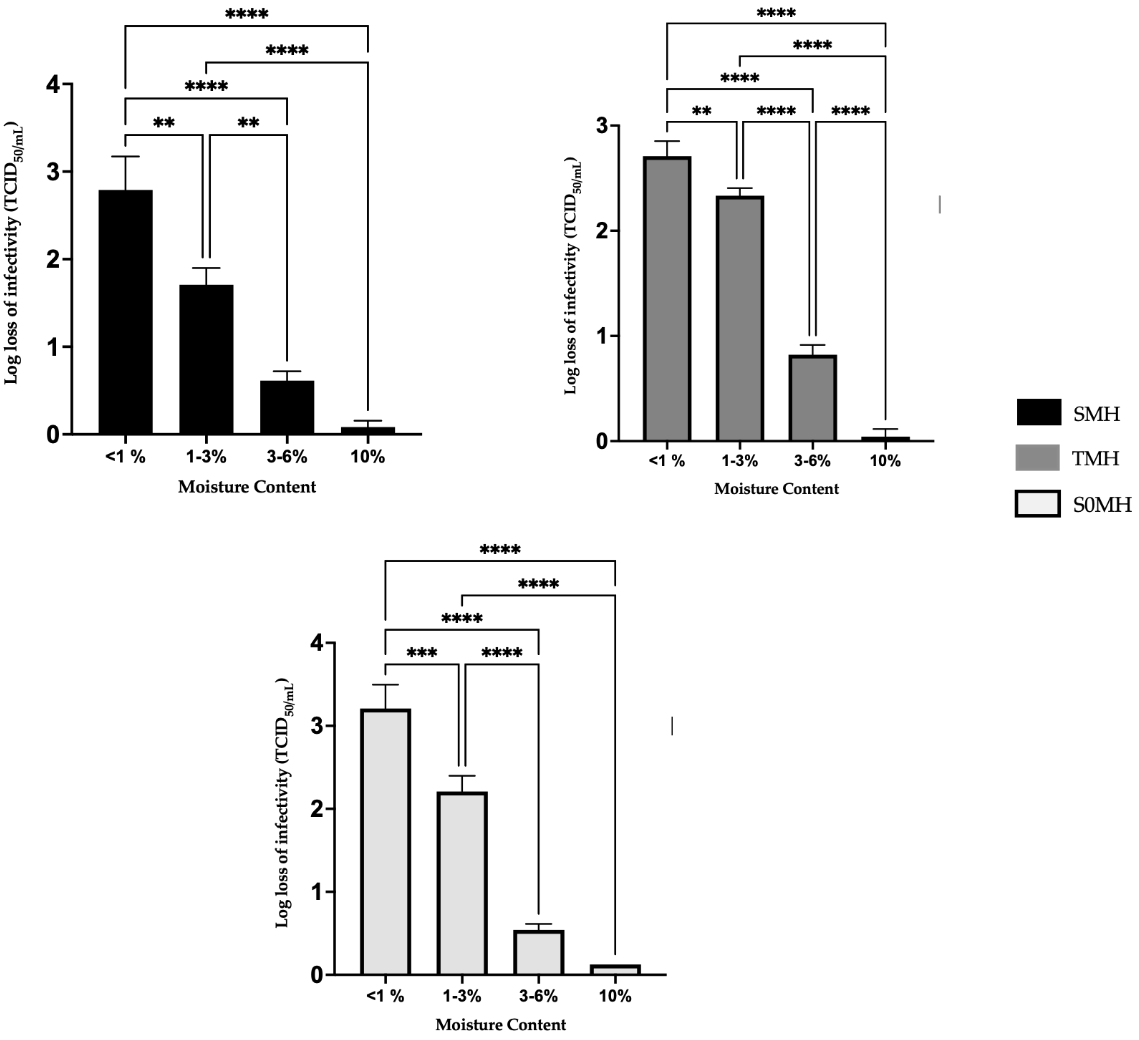

Supplementary Figure S6**.** Related to Figure 2 where one-way ANOVA analysis was performed between different moisture content groups of three formulations SMH, TMH and SoMH. The significant statistical difference is denoted through the asterisks (*) sign. Significant statistical differences were identified when the *p*-values were less than 0.05 (****$p < 0.0001$; *** $p < 0.001$; ** $p < 0.01$; * $p < 0.05$).

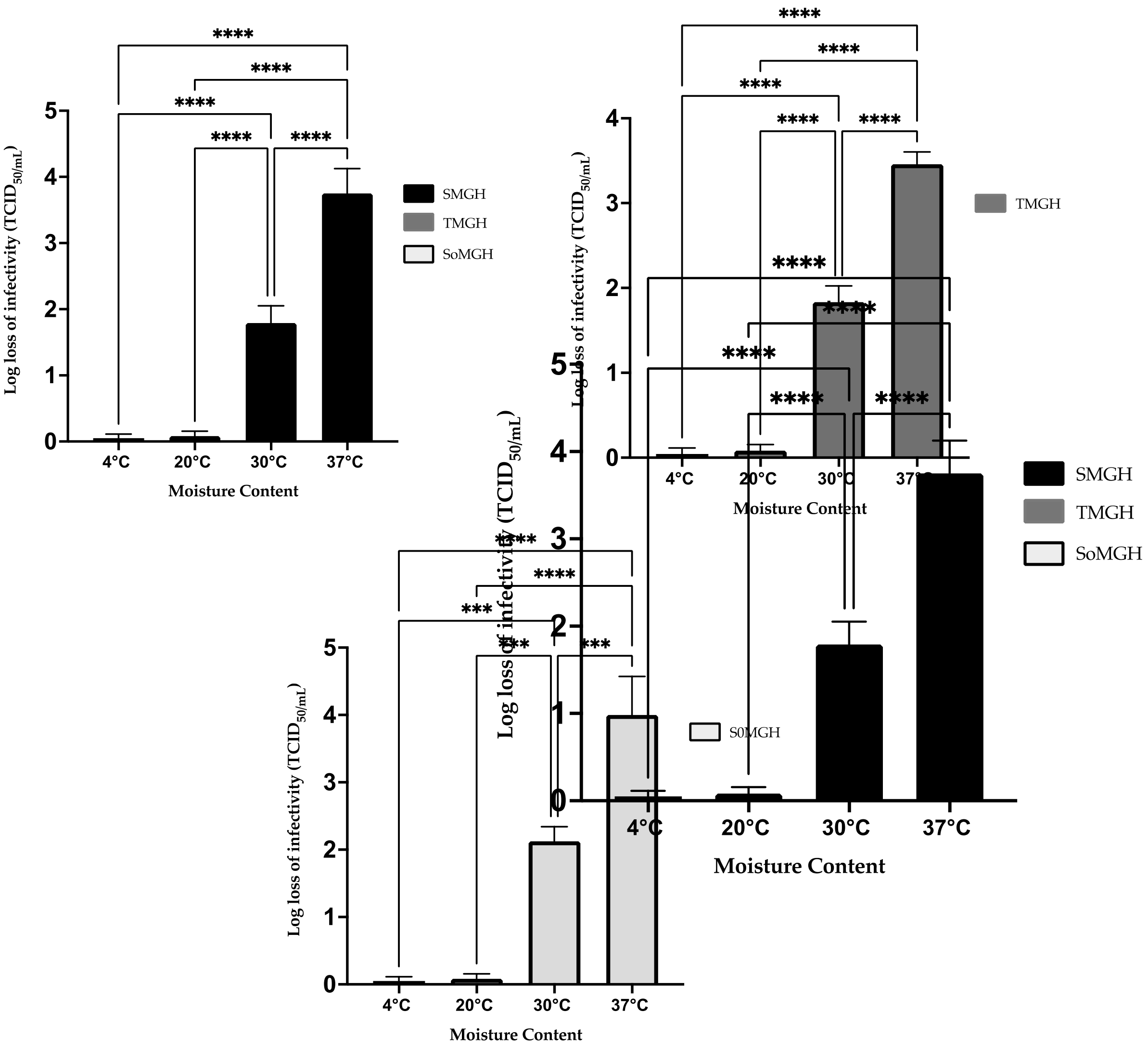

Supplementary Figure S7. Related to Figure 4 where one-way ANOVA analysis was performed between different temperature groups of three formulations SMGH, TMGH and SoMGH. The significant statistical difference is denoted through the asterisks (*) sign. Significant statistical differences were identified when the *p*-values were less than 0.05 (****$p < 0.0001$; *** $p < 0.001$; ** $p < 0.01$; * $p < 0.05$).

Supplementary Table S2. pH study of six different formulations before and after freeze-drying.

Formulation	Sample No	pH Before FD	pH After FD
**SMH**	Sample 1	7.53	7.25
	Sample 2	7.54	7.26
**TMH**	Sample 1	7.49	7.23
	Sample 2	7.55	7.27
**SoMH**	Sample 1	7.51	7.22
	Sample 2	7.55	7.24
**SMGH**	Sample 1	7.20	7.09
	Sample 2	7.26	7.07
**TMGH**	Sample 1	7.22	7.05
	Sample 2	7.26	7.07
**SoMGH**	Sample 1	7.21	7.06
	Sample 2	7.24	7.10

Reconstitution buffer: Phosphate buffer saline (pH: 7.22), Temperature Range: 22.4 ℃- 23.3 ℃, FD: Freeze-drying
